# Analysis of Influencing Factors on the Tribological Behavior of 42CrMo4/17NiCrMo6-4 under Grease Lubrication

**DOI:** 10.3390/ma16206699

**Published:** 2023-10-15

**Authors:** Fenghe Wu, Zhanpeng Jiang, Zijian Liu, Yingbing Sun, Xiang Li

**Affiliations:** Department of Mechanical Engineering, Yanshan University, Qinhuangdao 066004, China; risingwu@ysu.edu.cn (F.W.); jzp@stumail.ysu.edu.cn (Z.J.); liuzijian@ysu.edu.cn (Z.L.); lx@stumail.ysu.edu.cn (X.L.)

**Keywords:** tribological behavior, friction pair, hardness matching, wear mechanism

## Abstract

The tribological behavior of 42CrMo4/17NiCrMo6-4 under grease lubrication was explored in terms of load, speed, hardness matching, and lubrication quantity. Optical microscopy, scanning electron microscopy, and a surface profilometer were used to investigate the wear mechanism. The results show that hardness matching has the greatest impact on the wear resistance and friction reduction of the friction pair, followed by the load factor, with the impacts of speed and lubricant quantity being minor. Increasing the hardness of 42CrMo4 reduces the friction coefficient and wear volume of the friction pair substantially. When the maximum surface hardness of 42CrMo4 was compared with the lowest surface hardness, the friction coefficient was reduced by 21.5%, and the wear volume was reduced by 87.2%. Abrasive wear is the sort of wear failure that was seen, and as the hardness of 42CrMo4 increased, more severe fatigue wear appeared on 17NiCrMo6-4. While the wear volume initially increases and subsequently lowers with increasing load, the friction coefficient initially decreases and then stabilizes. A synergistic combination of abrasive and adhesive wear occurs under high load, changing the wear type from abrasive wear under low load. The wear volume is decreased by the sticky layer generated under high load conditions, which achieves superior wear prevention. This study is anticipated to offer recommendations for designing gears’ required hardness under various operating circumstances.

## 1. Introduction

**Figure 1:**
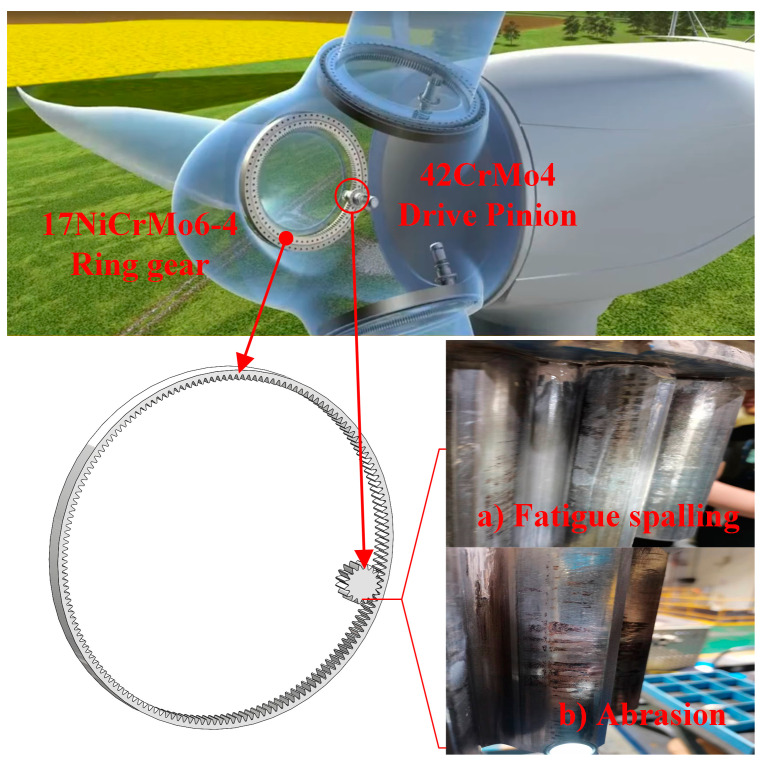
Wear in the pitch bearing gear pair and driving gear tooth surface of a wind turbine.

Due to its low cost per unit of electricity, green and ecologically favorable properties, and resource abundance, wind power generation has grown in importance within the worldwide renewable energy sector [1,2]. A wind turbine generator’s pitch control system is mounted between the hub and blades, as seen in Figure 1. It changes the pitch angle by rotating a tiny gear that meshes with an internal ring gear to change the blade angle. The small gear, however, engages with it more frequently within the same period because of the internal ring gear’s large gear ratio. The driving small gear is particularly vulnerable to excessive surface wear and fatigue spalling when relative sliding occurs at the contact surface [3,4]. As a result, it falls significantly short of the typical design life, causing a large annual loss in materials and components. Additionally, it significantly compromises the mechanical equipment’s safety [5,6]. In mountainous regions and close to the ocean, where they are impacted by geography and climate, wind turbines are frequently installed. These turbines’ maintenance cycles can run for several months. In addition to the loss of energy production during downtime, the wind turbine’s lifespan may also be further shortened by causes including micro-motion wear. Therefore, it is vital to assess the impact of various parameters on the friction performance of the pitch bearing-gear pair under grease lubrication circumstances to optimize the performance of the mating materials and meet the requirements for the stable operation of wind turbines.

The small driving gear in a pitch bearing-gear pair is typically constructed of 42CrMo4, while the mating ring gear is built of 17NiCrMo6-4. During operation, the contact surfaces of the gear pair wear continually [7,8]. During the operation of gear pairs of different material combinations, wearing limits the lifetime and compliance of the functionally relevant tolerances [9]. Previous studies determined the influence of the mechanical component properties and surface topography of the metal gear [10]. Hardness [11] and roughness [12] are of importance. In addition, model tests have identified the load resulting from surface pressure and sliding velocity as influencing variables [13].

Many researchers have investigated the frictional performance of 42CrMo4 in various conditions. Zhang et al. [14] studied the effect of CuNiAl on the torsional micromotion and sliding wear performance of 42CrMo4 under dry friction conditions. The experimental results revealed that when the load rose, the sliding friction coefficient reduced dramatically because of the solid lubricating effect of wear debris and wear-induced surface hardening. Liu et al. [15] investigated the frictional behavior of CuNiAl on 42CrMo4 under various lubrication conditions and discovered that wear was lightest in oil, most severe in dry friction circumstances, and corrosion products reduced friction and wear in seawater lubrication. Researchers are currently working to improve the friction and wear performance of 42CrMo4 through surface strengthening [16,17,18,19], heat treatment [20], surface texturing, and lubricant modification [15,21,22]. Many surface modification approaches, however, continue to struggle to handle the demanding operating requirements of wind turbine gears, such as strong transient loads, unstable speeds, and huge temperature changes.

A few research projects have already investigated the influence of various component properties of the metallic partner on the wear behavior of the material pairing. Research work is carried out both on gear pairings [12] as well as on model systems such as pin-on-disk [23] or two-disk test rigs. The influence of hardness when applying high-strength steel materials was investigated by Wieleba [11], and reduced wear was found at very high hardness. To date, there has been no targeted investigation of the influence of the hardness of the metallic pinion and the resulting application limits for different metallic materials. 42CrMo4 and 17NiCrMo6-4 are commonly used materials for wind turbine bearing-gear pairs. The material heat treatment process is prone to the issue of unstable hardness values, but no researchers have specifically studied the wear characteristics of this mating material under different operating conditions. The appropriate hardness combination is of great significance for optimizing the wear performance and overall lifespan of the friction pair, reducing safety hazards, and lowering maintenance costs.

This study aims to examine the mechanical properties and application limits of heat-treated metal small gears in the 42CrMo4/17NiCrMo6-4 material pairing. The primary focus is to investigate the impact of hardness matching on the wear mechanism. To achieve this, the hardness of 42CrMo4 specimens was modified using heat treatment. Wear tests were conducted to analyze the specific wear behavior of the material and determine the wear mechanism and application limits of metal materials.

This study addresses the issues of the harsh operating environments of wind turbines and the instability of hardness values during the heat treatment process of gear materials. The study investigates the effects of grease lubrication, load, speed, hardness ratio, and lubricant quantity on the friction and wear performance of 42CrMo4/17NiCrMo6-4 systems using orthogonal experiments. It is observed that lubricating grease plays a significant role in improving the friction and wear performance of these materials, particularly under low-speed, heavy-load conditions, with a notable effect [24]. Building upon the significance analysis, a more comprehensive analysis is conducted to examine the influence of different loads and hardness ratios on the friction coefficient, wear volume, and wear mechanisms. This research contributes to expanding the available data in this research field and provides guidance for gear design and the selection of operating conditions, ultimately enhancing the service life of gear pairs.

## 2. Specimen Preparation and Experimental Methods

### 2.1. Specimen Preparation

The experimental materials were selected based on wind turbine gear pair materials. For the upper specimen, 17NiCrMo6-4 was directly cut from the gear shaft using electrical discharge wire cutting. The spherical head was machined using CNC turning, and the real heat treatment process of carburizing and quenching, representative of wind turbine gears, was applied to ensure a surface hardness greater than 60 HRC. After polishing, the desired upper specimen with 17NiCrMo6-4 was obtained.

**Figure 2:**
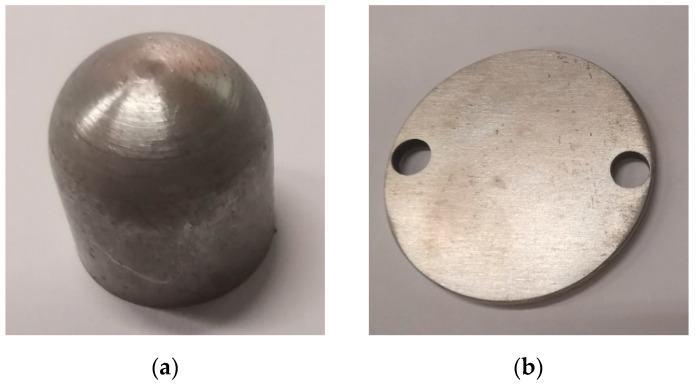
Test specimens. (**a**) upper specimen with 17NiCrMo6-4; (**b**) lower specimen with 42CrMo4.

For the lower specimen, 42CrMo4 was selected and processed into a disc shape. Initially, the specimen was machined to the desired dimensions, and then different hardness values for the required tests were achieved by adjusting various heat treatment process parameters. To ensure high strength and toughness in the gear core, the material underwent initial quenching and subsequent low-temperature tempering at 900 °C oil quenching followed by 150–180 °C tempering. This process resulted in a higher hardness tempered martensitic structure on the surface, with a maximum hardness of 60 HRC. Additionally, the material was subjected to 840 °C oil quenching and 470 °C medium-temperature tempering, which yielded a lower hardness tempered bainitic structure with a hardness of 44 HRC. The chemical composition of the material is provided in Table 1 and Table 2. The actual processed specimens are shown in Figure 2.

The lubricating grease selection brand is AG14-61, which is suitable for high and low-temperature applications. It is suitable for the temperature range of −50~120 °C and has excellent adhesion, which can not only improve the wear resistance of gears but also prevent the corrosion of parts. The grease characteristics of AG14-61 are provided in Table 3.

### 2.2. Characterization and Analysis

The worn 42CrMo4/17NiCrMo6-4 were characterized for wear volume using a three-dimensional non-contact surface profilometer (Newview 9000, ZYGO, Santa Clara, CA, USA) to observe the three-dimensional micro-topography. The white light interferometry principle was employed to measure the worn area on the surface of 42CrMo4, obtaining depth profiles of wear scars at multiple locations. Upon integrating these profiles, the wear cross-sectional area was determined. Taking the average value of the cross-sectional area and multiplying it by the wear circumference yielded the wear volume, as shown in Equation (1).

(1) $$S=\int_{a}^{b}f\left(x\right)dx,$$

where $f\left(x\right)$ is wear scar curve function, in mm; S is calculate cross-sectional area, in ${\mathrm{mm}}^{2}$; a, b are starting and ending points of wear, dimensionless.

For the calculation of the wear volume on the spherical head with 17NiCrMo6-4, the length of the worn surface was measured using the surface profilometer. The wear volume was then obtained by converting the measured length using the formula for the volume of a spherical segment, as shown in Equation (2).

(2) $$V=\frac{\pi }{3}\left(2r+\sqrt{r^{2}-a^{2}}\right)\cdot {\left(r-\sqrt{r^{2}-a^{2}}\right)}^{2},$$

where V is the wear volume of the upper sample, in ${\mathrm{mm}}^{3}$; r is the radius of the upper sample ball head, in mm; a is the radius of the worn circular surface of the upper sample, in mm;

The surfaces of the worn samples were analyzed with micro-topography using the Thermo Scientific™ (Waltham, MA, USA) Verios G4 field emission scanning electron microscope (SEM) introduced by FEI Company in the United States. The SEM offers a resolution exceeding 0.8 nm. By focusing an electron beam onto the sample surface, the SEM enables interactions between the electron beam and different atoms in the sample, resulting in surface topography and composition signals. To ensure the electron beam remains undistorted and to enhance image quality, the entire testing environment was maintained under vacuum conditions, facilitating a more accurate determination of wear types and enabling the analysis of wear mechanisms.

### 2.3. Experimental Methods

**Figure 3:**
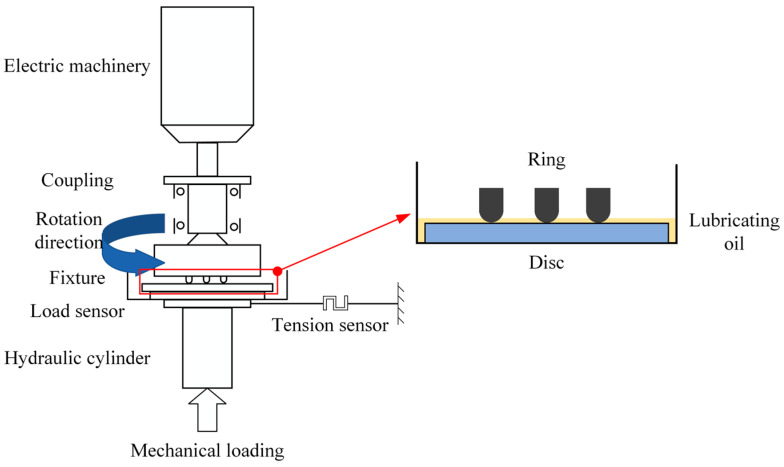
Schematic diagram of the experimental machine.

The MDW-5G friction and wear test machine adopts a disc configuration for tribological experiments. The friction specimens are fixed onto the freely rotating test table through holes, and temperature, friction force, and friction coefficient are measured in real time using force and temperature sensors installed on the test table. The counterpart specimens are fixed onto a fixture mounted on the spindle using screw holes and side holes. The test machine’s hydraulic system applies the load. The schematic diagram of the experimental setup is shown in Figure 3.

Considering the actual transmission conditions of the driving pinion and inner ring gear, Equation (3) calculates the contact stress as 187.5 KN based on the torque and gear radius.

(3) $$T=\frac{9.55Fr}{n},$$

where T is rated torque, in N⋅m; F is contact load, in N; n is gear speed, in r/min.

Utilizing the relationship between contact load, gear width, contact radius, elastic modulus, and Poisson’s ratio, the contact stress on the gear surface can be accurately determined with Equation (4), resulting in 1.04 GPa, the equivalent load is approximately 1000 N.

(4) $$\sigma =\sqrt{\frac{F}{\pi b}\left(\frac{\frac{1}{\rho }}{\frac{1-\mu_{1}^{2}}{E_{1}}+\frac{1-\mu_{2}^{2}}{E^{2}}}\right)},$$

where σ is contact stress, in MPa; F is contact load, in N; b is gear width, in m; ρ is gear contact radius, in m; E is elastic modulus, in MPa; μ is Poisson’s ratio, dimensionless parameter.

To calculate the sliding speed of the gear, we consider the angular speeds of the pitch at 2°/s, 3°/s, 4°/s, 5°/s, and 6°/s. It is important to note that the sliding rate of the gear mesh is zero at the pitch circle, while the sliding coefficient reaches its maximum at the extreme meshing point. The sliding coefficient varies depending on the position of the meshing point. By utilizing Equation (5), we can determine the relative sliding speed. Finally, we find that the speed of the testing machine should be set between 50–170 r/min.

(5) vtt=w1i×N1N−2w1i+w1×KN1,

where $N_{1}N_{2}$ is theoretical meshing line length, in m; $KN_{1}$ is sliding coefficient; i is transmission ratio, dimensionless; $v_{tt}$ is relative sliding speed, in m/s; $w_{1}$ is driving gear angular velocity, in rad/s.

**Figure 4:**
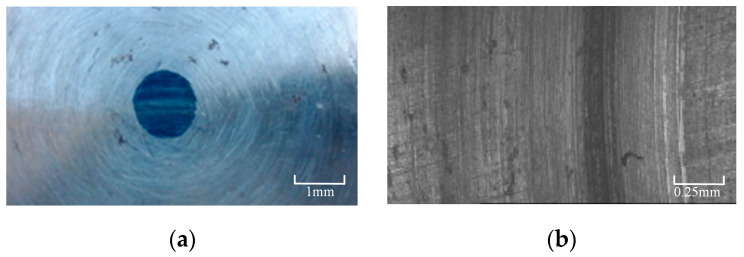
Preliminary test specimens under 52–60 HRC—600 N—100 r/min–36,000 r. (**a**) upper specimen with 17NiCrMo6-4; (**b**) lower specimen with 42CrMo4.

Since it is not possible to simulate friction and wear tests under grease lubrication conditions, the selection of specific test parameters requires preliminary preparatory tests. For the 42CrMo4 sample with a hardness of 52 HRC, the following parameters were chosen: a rotational speed of 100 r/min, a load of 600 N, a wear time of 6 h, and a total test revolution of 36,000 r. The specimen after the experiment is shown in Figure 4.

**Figure 5:**
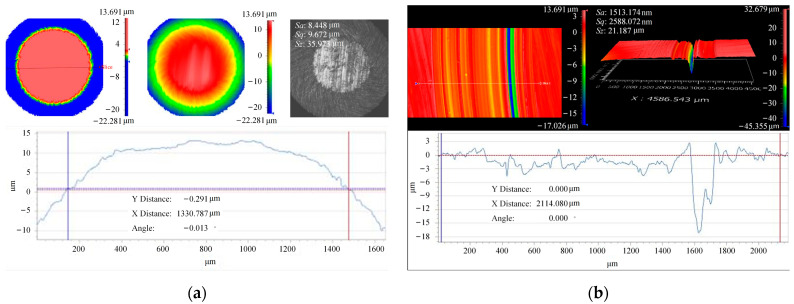
Wear map under 52–60 HRC—600 N—100 r/min—36,000 r. (**a**) upper specimen with 17NiCrMo6-4; (**b**) lower specimen with 42CrMo4.

Figure 5 shows the wear surface morphology, exhibiting clear wear marks up to a depth of 20 μm in the severely worn area and an average depth of 3–4 μm. The three-dimensional morphology of the wear ball in the upper sample indicates a wear diameter of 1.4 mm. Based on the preliminary tests, it was observed that the contact area of the ball disc does not significantly increase with wear time. Therefore, a contact area of 1 ${\mathrm{mm}}^{2}$ was chosen for the test, considering the actual working conditions of the gear. The final selected test load ranged from 400–1200 N, ensuring that the initial contact stress during wear exceeds the actual contact stress of the gear. As wear progresses and the contact area increases, the contact stress approaches the actual contact stress until it becomes lower, covering the actual working condition. To maintain consistency with the experimental parameters and actual working conditions and to prevent lubricating grease difficulty at excessively high rotational speeds, the maximum rotational speed was not increased. Taking the experiment duration into account, the total number of revolutions was appropriately reduced to 20,000 r (equivalent to a sliding distance of 1446 m).

In terms of hardness selection for 42CrMo4, considering specific heat treatment processes, the surface hardness can reach a maximum value of around 60 HRC(700 HV). The final selected hardness ratios are 60–44 HRC, 60–48 HRC, 60–52 HRC, 60–56 HRC, and 60–60 HRC. Among them, the upper specimen (17NiCrMo6-4) maintains a constant hardness of 60 HRC.

The lubrication range is determined based on the existing lubrication thickness, considering both excessive and insufficient lubrication. Therefore, we determine five gradients as 0.5 mm, 1 mm, 1.5 mm, 2 mm, and 2.5 mm.

The friction coefficient is automatically recorded by the test machine. All tests are repeated three times, and the average values are taken from the obtained data to ensure stability and accuracy. The experimental parameter settings are shown in Table 4.

## 3. Results and Discussion

### 3.1. Significance Analysis

#### 3.1.1. Friction Coefficient

**Figure 6:**
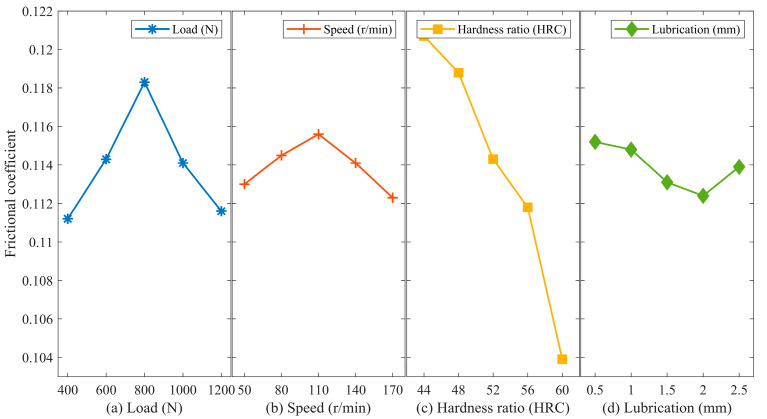
Main effect analysis of friction coefficient. (**a**) Load; (**b**) Speed; (**c**) Hardness ratio; (**d**) Lubrication.

Figure 6a demonstrates that the friction coefficient initially increases and then decreases with an increase in load. The initial stage of the friction coefficient’s increase is due to the greater involvement of micro-asperities within the friction system as the load increases. However, in the later stage, as the load continues to increase, the friction coefficient decreases.

Figure 6b indicates that the friction coefficient exhibits an increasing trend followed by a decreasing trend with an increase in sliding velocity. The friction coefficient reaches its maximum value at an intermediate velocity. A decrease in the friction coefficient at high rotational speeds may be attributable to the increased temperature and generation of more base oil, as well as the enhanced effect of hydrodynamic lubrication at higher speeds. Therefore, when considering friction reduction, it is advisable to choose lower or higher rotational speeds.

According to Figure 6c, the friction coefficient decreases with an increase in the hardness of the lower specimen, and the effect is most pronounced. A higher hardness leads to better friction reduction, indicating that an increase in hardness significantly contributes to reducing the friction coefficient.

Furthermore, Figure 6d shows that with an increase in lubricant quantity, the friction coefficient initially decreases and then increases. The influence of lubricant quantity on the friction coefficient is relatively small. According to the EHD (Elasotohydrodynamic) theory, the experimental results are consistent with the Stribeak curve [25]. During the mixed lubrication stage, the penetration of lubricating oil gradually reduces the friction coefficient. However, after this stage, the viscosity of the lubricating oil comes into play, leading to an increase in the friction coefficient. Consequently, the application of excessive lubricant grease does not improve the anti-wear performance of the friction pair.

#### 3.1.2. Wear Volume of 17NiCrMo6-4

**Figure 7:**
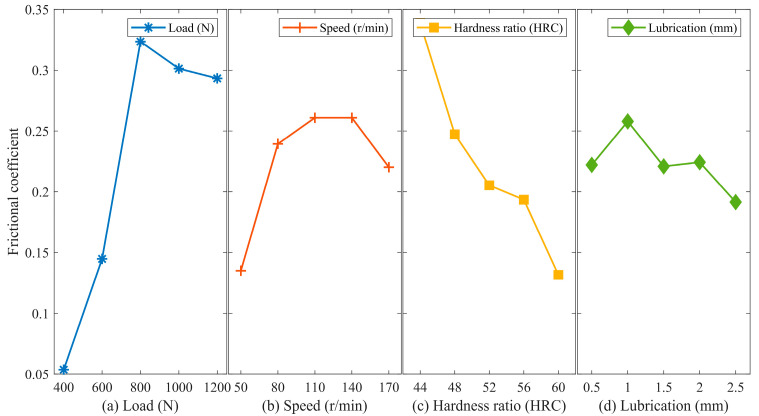
Main effect analysis of 17NiCrMo6-4 wear volume. (**a**) Load; (**b**) Speed; (**c**) Hardness ratio; (**d**) Lubrication.

According to Figure 7a, the wear volume of 17NiCrMo6-4 initially increases and then decreases as the load increases. The initial stage shows a significant and strongest increase, but under high-load conditions, the wear volume decreases. This indicates a change in the wear mechanism. From a design perspective, it is advisable to minimize the actual load as much as possible.

As shown in Figure 7b, the wear volume of 17NiCrMo6-4 exhibits an increasing trend followed by a decreasing trend as the sliding velocity increases. The wear volume is relatively small at low and high sliding velocities. At low sliding velocities, the increase in velocity leads to reduced lubricant flowability, resulting in a decreased amount of lubricant replenishing the worn surface within a unit of time. This leads to a poorer quality of lubricating film after the breakdown of the oil film, resulting in increased wear volume. However, as the sliding velocity continues to increase, a higher sliding velocity generates more base oil and enhances the hydrodynamic lubrication effect. This facilitates the formation of a stable oil film capable of withstanding higher pressure and reduces the contact area on the friction pair surfaces, resulting in improved anti-wear properties.

Figure 7c demonstrates that as the hardness of 42CrMo4 increases, the wear volume of 17NiCrMo6-4 decreases. This effect is significant, and the decreasing trend is relatively uniform. This indicates that increasing the hardness of 42CrMo4 significantly improves the wear resistance of the friction pair.

Figure 7d illustrates that with an increase in lubricant quantity, the wear volume of 17NiCrMo6-4 initially increases and then slightly decreases, but the change trend is not significant. The minimum wear volume is observed at a lubricant quantity of 2.5 mm, with a small range of variation (0.0664). This indicates that the lubricating grease provides effective anti-wear properties under all lubrication conditions.

In summary, the analysis reveals that the magnitude of the load has the most significant impact on the wear volume, followed by hardness matching and sliding velocity. The impact of lubricant quantity is relatively minimal.

#### 3.1.3. Wear Volume of 42CrMo4

**Figure 8:**
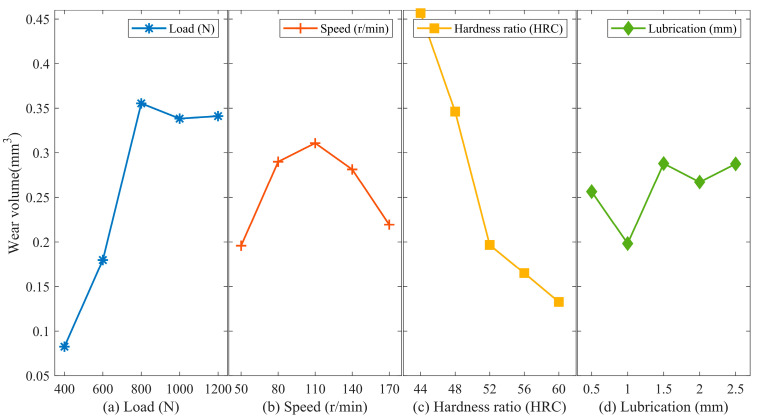
Main effect analysis for wear volume of 42CrMo4. (**a**) Load; (**b**) Speed; (**c**) Hardness ratio; (**d**) Lubrication.

According to Figure 8a, the wear volume of 42CrMo4 initially increases and then stabilizes as the load increases. There is a significant increase in wear volume when the load increases from 600 N to 800 N, consistent with the rate of change in wear volume of 17NiCrMo6-4. Therefore, it is advisable to avoid intermediate load contacts as much as possible.

As shown in Figure 8b, the wear volume of 42CrMo4 exhibits an increasing trend followed by a decreasing trend as the sliding velocity increases. The smallest wear volume is observed at the highest sliding velocity. The explanation for this trend is the same as the one provided in the analysis of the rate of change in wear volume of 17NiCrMo6-4 in Section 3.1.2.

Figure 8c demonstrates that as the hardness of 42CrMo4 increases, the wear volume of 42CrMo4 decreases. The effect is significant, with the most significant decrease observed when the hardness of 42CrMo4 increases from 48 HRC to 52 HRC. Therefore, it is important to ensure a higher hardness for 42CrMo4 in practical engineering applications.

As depicted in Figure 8d, the wear volume of 42CrMo4 fluctuates within a small range as the lubricant quantity increases. This confirms the weak influence of lubricant quantity variation on the wear volume, as stated in the experimental design. The lubricating grease color changes from milky white to gray after wear, which is due to the inclusion of wear debris in the lubricating grease during the wear process.

The analysis indicates that hardness matching has the most significant impact on the wear resistance of 42CrMo4, followed by load, sliding velocity, and lubricant quantity. The impact of these factors on the wear volume of 42CrMo4 is consistent with that of 17NiCrMo6-4.

### 3.2. Univariate Analysis

Based on the analysis, it is evident that load and hardness matching have a significant impact. Therefore, this study focuses on analyzing and discussing the effects of these two factors on friction and wear. The following experimental plans have been formulated:

Experiment 1: In the main effect analysis, the influence of load on friction and wear performance was found to be second only to hardness matching. Hence, Experiment 2 was conducted to study the effects of load on friction and wear performance. The load values chosen were consistent with the orthogonal experimental parameters and were set as 400 N, 600 N, 800 N, 1000 N, and 1200 N. The hardness matching selected was the highest wear-resistant combination of 60–60 HRC. The speed was set at the intermediate value of 90 r/min, the lubricant film thickness was 1.5 mm, and the starting temperature was ambient temperature. The effects of different load conditions on the friction and wear performance of the mating pair were analyzed. The single-factor parameter settings for Experiment 1 are shown in Table 5.

Experiment 2: Hardness matching has the most significant influence on friction and wear performance. Hence, Experiment 2 investigates the impact of hardness matching on friction and wear performance. Five hardness gradients (44, 48, 52, 56, and 60 HRC) were determined for 42CrMo4 based on heat treatment processes. The load and speed were set at their respective intermediate values of 800 N and 90 r/min. The lubricant film thickness was 1.5 mm, and the starting temperature was room temperature. The effects of different hardness ratios on the friction and wear performance of the mating pair were analyzed. The single-factor parameter settings for Experiment 2 are shown in Table 6.

#### 3.2.1. Load

1. The influence of load on the friction coefficient

**Figure 9:**
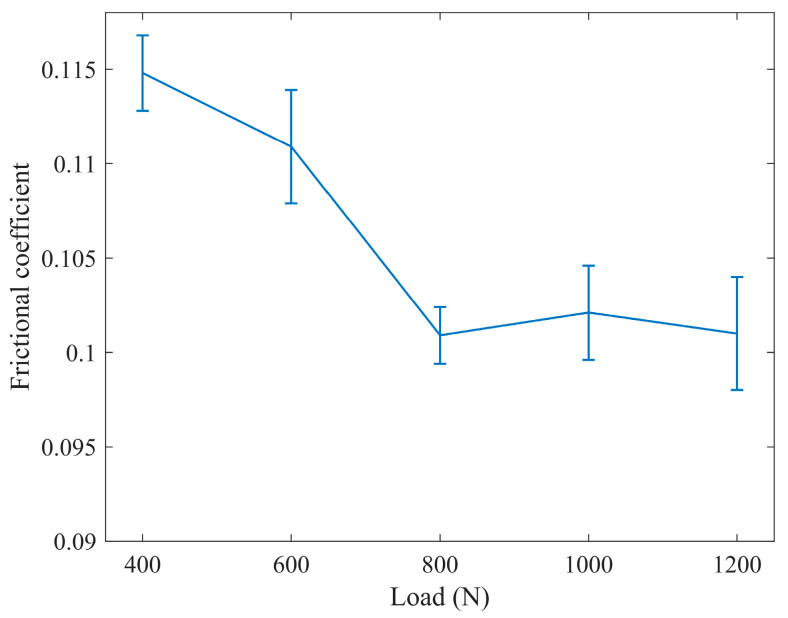
Friction coefficient diagram under different loads.

As shown in Figure 9, when the load is less than 800 N, the friction coefficient decreases with increasing load, and the decrease is relatively significant. When the load exceeds 800 N, the friction coefficient remains stable with increasing load, indicating that the friction coefficient is more sensitive to load variations at lower loads. When the load exceeds 800 N, the average friction coefficient stabilizes at around 0.101, indicating that it is beneficial to reduce the friction coefficient under higher loads.

Therefore, under the given experimental conditions, as the load increases from 400 N to 800 N, the friction coefficient decreases. This is because the surface of the specimens is composed of numerous micro-asperities, and the contact between the mating surfaces is essentially the contact between these micro-asperities. With an increase in load, more micro-asperities participate in the friction system. This is because, on the one hand, the actual contact area increases at a slower rate compared with the increase in load, resulting in a decrease in the friction coefficient. On the other hand, as the load increases, the heat generated by friction is much greater than the heat dissipated to the environment, leading to an overall increase in the temperature of the friction pair and lubrication environment. Additionally, the shearing thinning effect of the lubricating oil allows for the separation of more base oil in the lubricating grease, which can better replenish the friction pair in the wear area [26,27], resulting in improved lubrication and a reduction in the friction coefficient.

When the load exceeds 800 N, the friction coefficient stabilizes. This may be attributable to the fact that with the increase in temperature, the viscosity of the base oil decreases, making the lubricating film more prone to breakdown and reducing its load-carrying capacity. This leads to direct contact between more micro-asperities, thereby weakening the ability of the oil film to reduce the friction coefficient [28,29].

2. The influence of load on wear volume

**Figure 10:**
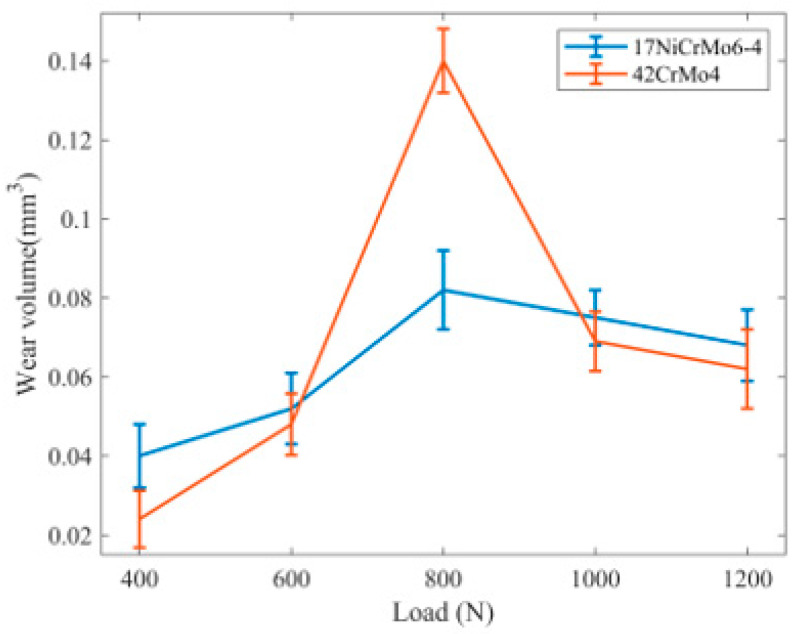
Wear volume diagram under different loads.

As shown in Figure 10, with an increase in load, the volume of wear exhibits a trend of initially increasing and then decreasing. The 17NiCrMo6-4 material shows a relatively stable trend, with the volume of wear maintained between 0.04~0.08 ${\mathrm{mm}}^{3}$. On the other hand, 42CrMo4 shows a greater variation, with the volume of wear ranging from 0.02 ${\mathrm{mm}}^{3}$ to 0.11 ${\mathrm{mm}}^{3}$.

For loads below 600 N and above 1000 N, 42CrMo4 demonstrates higher wear resistance, indicating a relatively smaller volume of wear. However, for loads between 600 N and 800 N, 17NiCrMo6-4 exhibits higher wear resistance, resulting in a relatively smaller volume of wear. Therefore, in gear design, it is advisable to avoid contact stresses being within the intermediate range.

**Figure 11:**
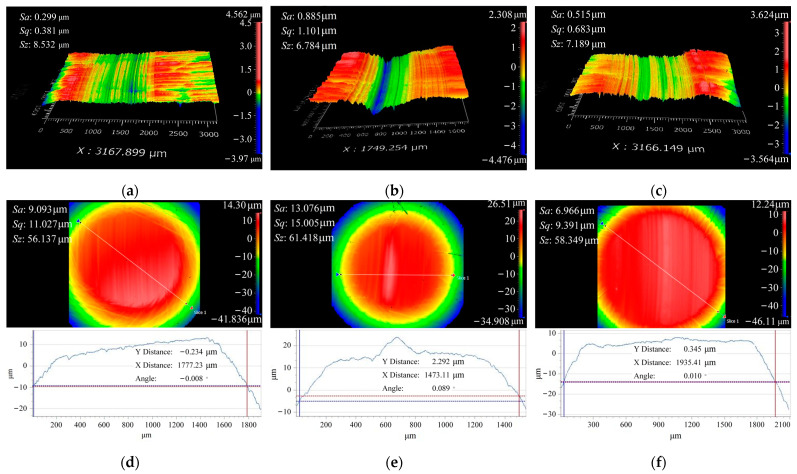
Surface topography under different loads. (**a**) 42CrMo4 under 400 N; (**b**) 42CrMo4 under 800 N; (**c**) 42CrMo4 under 1200 N; (**d**) 17NiCrMo6-4 under 400 N; (**e**) 17NiCrMo6-4 under 800 N; (**f**) 17NiCrMo6-4 under 1200 N.

**Figure 12:**
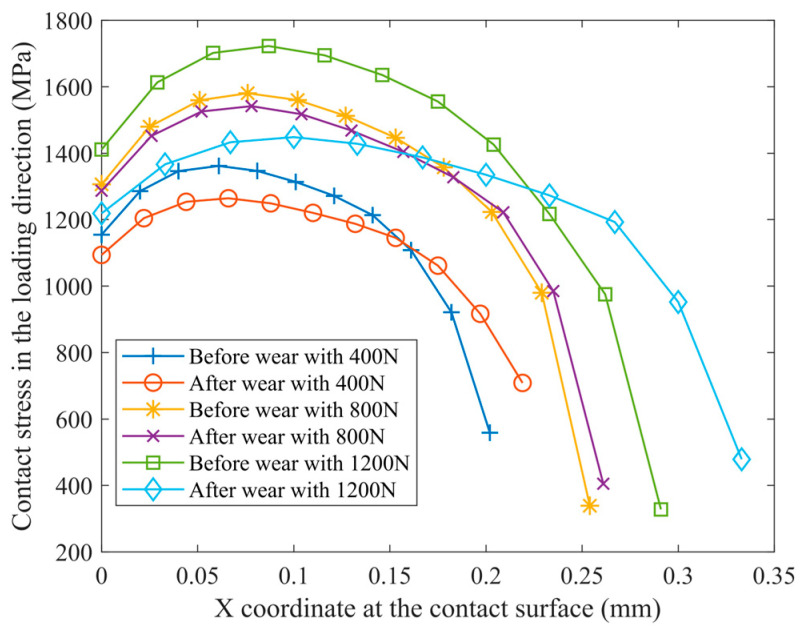
Stress in the loading direction along the contact interface before and after wear.

The surface topographies under the conditions of 400 N, 800 N, and 1200 N loads are shown in Figure 11. It can be observed that the wear severity is the lightest in Figure 11a), with only slight wear marks visible. The wear marks cover the surface grinding marks, and the depth of the wear marks is approximately 1 μm. As shown in Figure 12, during the progression of wear, the contact area of the friction pair increases in comparison to the initial contact area. This leads to a more uniform distribution of contact stress, resulting in a decrease in maximum stress and an increase in minimum stress. Consequently, the stress gradient is reduced. When the load is 800 N, the wear severity is the highest, with the maximum depth of wear marks in the central region reaching 4 μm. The contact area does not show a significant increase before and after wear, and the distribution of contact stress remains unchanged from the initial state. The severity of wear intensifies due to the application of high loads [30]. Under a load of 1200 N, the wear severity is relatively lighter, with the maximum depth of wear marks being approximately 2 μm. After undergoing wear, the contact area of the specimen reaches a maximum, aligning with the experimental observations of contact stress distribution under a load of 400 N. When the load exceeds 800 N and continues to increase, an intriguing phenomenon occurs: the wear volume decreases. This behavior is accompanied by the surface roughness pattern $S_{q}$ of 42CrMo4/17NiCrMo6-4, which shows consistency with the observed wear volume. This phenomenon may be attributable to a transition in the type of wear, and the specific wear mechanisms will be analyzed in conjunction with the micro-topography of the worn surfaces in subsequent analyses.

3. The influence of load on the surface microstructure

**Figure 13:**
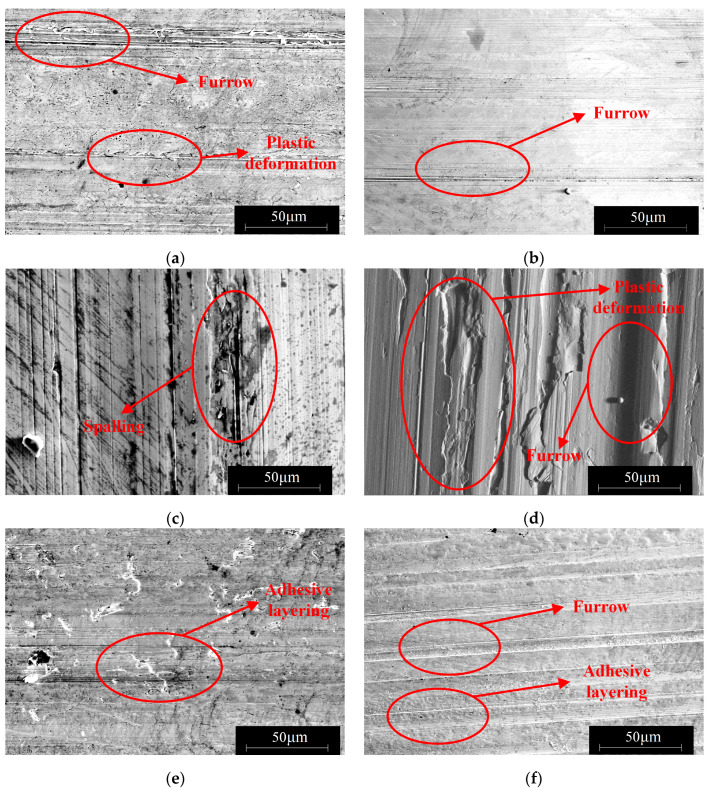
The surface microstructure under different loads. (**a**) 17NiCrMo6-4 under 400 N; (**b**) 42CrMo4 under 400 N; (**c**) 17NiCrMo6-4 under 800 N; (**d**) 42CrMo4 under 800 N; (**e**) 17NiCrMo6-4 under 1200 N; (**f**) 42CrMo4 under1200 N.

**Figure 14:**
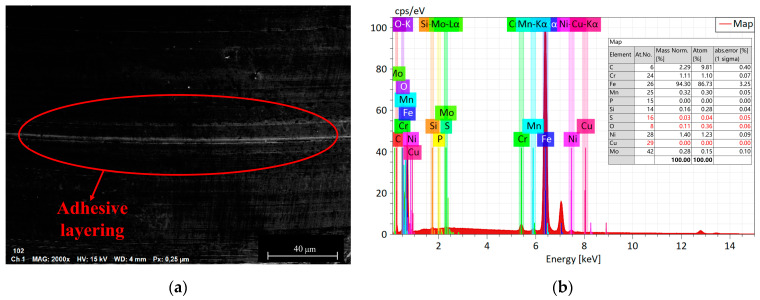
Wear morphologies and EDS diagrams of 42CrMo4 at applied loads of 800 N. (**a**) the wear morphologies of 42CrMo4; (**b**) EDS diagrams of 42CrMo4.

**Figure 15:**
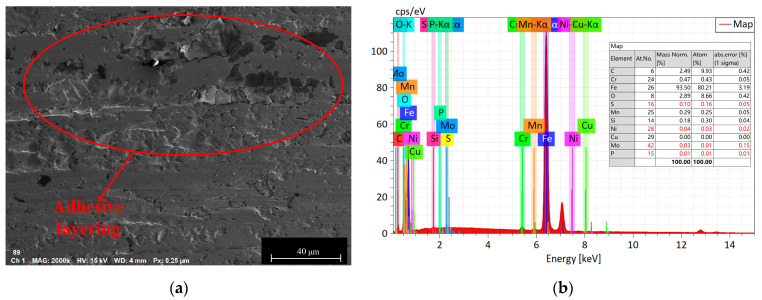
Wear morphologies and EDS diagrams of 17NiCrMo6-4 at applied loads of 800 N. (**a**) the wear morphologies of 17NiCrMo6-4; (**b**) EDS diagrams of 17NiCrMo6-4.

The influence of load on the surface microstructure is evident in the surface topographies of specimens subjected to 400 N and 1200 N loads, as shown in Figure 13. By comparing these with the surface morphology under an 800 N load, depicted in Figure 11b, the following observations can be made:

- When the load is below 800 N, the predominant wear type on 42CrMo4 is abrasive wear [31], accompanied by a small amount of plastic deformation. This can be attributable to the slight plastic deformation of the contact asperities on the surface when the load is relatively low, while fewer asperities actively participate in the frictional process. As a result, the abrasive particles generate fewer grooves along the sliding direction on the material surface. However, as the load increases, the plastic deformation of the asperities intensifies, involving a greater number of asperities in the frictional process, leading to deeper grooves and increased surface wear. Furthermore, the increase in load generates higher frictional heat, causing the matrix to soften, which promotes adhesive wear and contributes to an overall increase in wear volume, as shown in Figure 14.
- At an 800 N load, 17NiCrMo6-4 experiences severe spalling. This is attributable to the higher cyclic stress exerted on the surface of the specimen. With the same number of cycles, the likelihood of cracks forming becomes higher, and under greater pressure conditions, spalling occurs from the crack sites due to the tearing effect of adhesive wear [32], as shown in Figure 15.
- Based on the three-dimensional topography (Figure 11c) and microtopography images (Figure 13e,f) under a 1200 N load, it can be observed that the predominant wear types on 42CrMo4 are abrasive wear and adhesive delamination [31], while 17NiCrMo6-4 exhibits primarily plastic flow and adhesive delamination. Even after ultrasonic cleaning, a layered structure is still visible. This is attributable to the excessively high load, which generates a significant amount of wear debris in the early stage of wear. Some of the debris escapes the frictional system, while the remaining debris undergoes repeated compaction on the material surface under high loads. The high shear stress induces severe plastic deformation between the specimens, resulting in welding phenomena. The surface hardness is further increased due to the work-hardening effect. The presence of an adhesive layer on the specimen surface hinders the initiation of new wear, providing better protection for the underlying substrate. As a result, the wear volume decreases with increasing load.

#### 3.2.2. Hardness Matching

1. The Influence of hardness matching on friction coefficient

**Figure 16:**
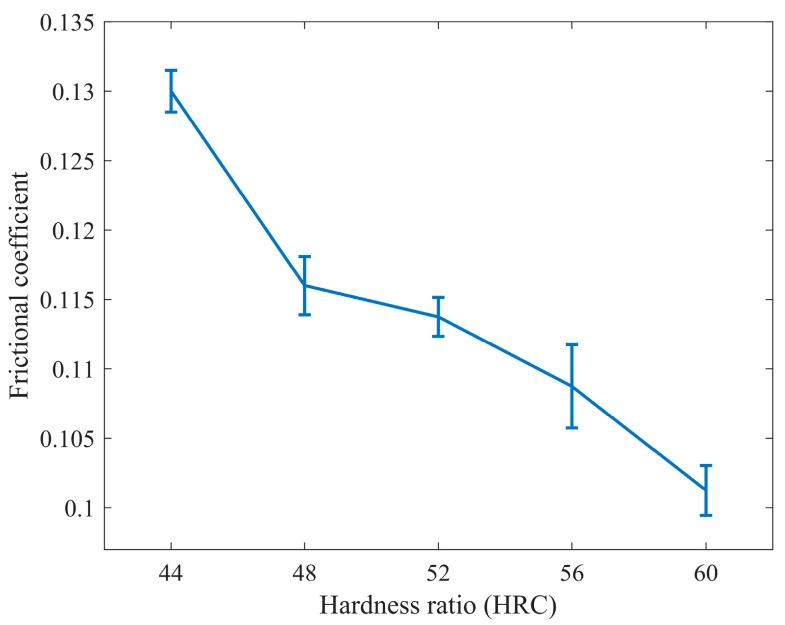
Friction coefficient diagram under different hardness matching.

As shown in Figure 16, the average friction coefficient decreases with increasing surface hardness. The trend of change is more significant in the initial stage, relatively moderate in the middle section, and becomes prominent again when the hardness exceeds 56 HRC. This indicates that the increase in hardness has a more pronounced effect on reducing the friction coefficient in the early and later stages.

In cases where there is a significant difference in hardness between the friction pair, the surface asperities of the harder specimen are more likely to penetrate the surface of the softer specimen, resulting in an increased plowing effect. As hardness increases, the surface asperities of the specimen become harder. Under a certain load, when relative motion occurs between the friction pair, it becomes difficult for the asperities of the specimen to penetrate the surface of the mating specimen. As a result, fewer asperities penetrate the surface of the mating specimen, leading to reduced resistance and a decrease in the friction coefficient. Additionally, the increase in hardness may cause a transition in the wear mechanism, making it more difficult for abrasive wear to occur due to the reduced penetration of the friction pair into the test specimen. A lower friction coefficient results in reduced friction force and can minimize friction losses, sustainably reduce noise under high-speed conditions, and greatly improve the working condition of gear pairs, such as driving gears and gear rings, ensuring their long-term normal operation. The results indicate that increasing the hardness ratio of the specimen surfaces can effectively reduce the friction coefficient, minimize wear, and extend the service life of wind turbine gears.

2. The Influence of hardness matching on wear volume

**Figure 17:**
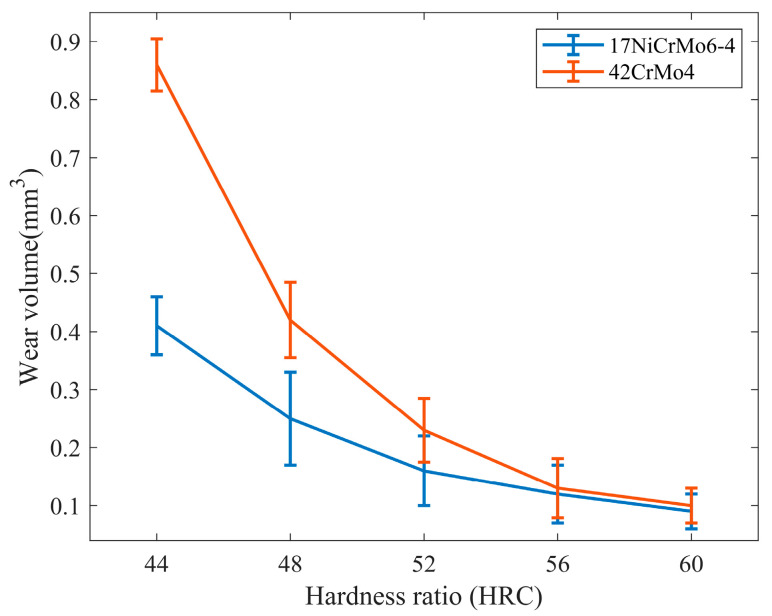
Wear volume diagram under different hardness matching.

**Figure 18:**
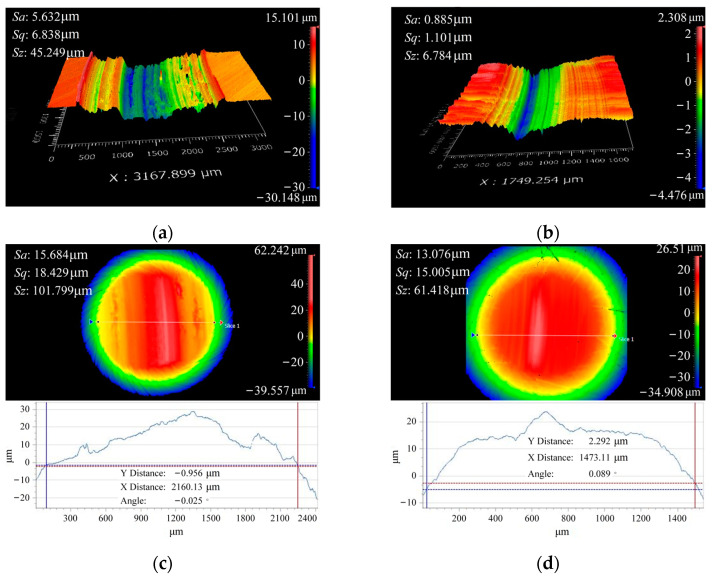
Surface topography under different loads. (**a**) 42CrMo4 with 44–60 HRC; (**b**) 42CrMo4 with 60–60 HRC; (**c**) 17NiCrMo6-4 with 44–60 HRC; (**d**) 17NiCrMo6-4 with 60–60 HRC.

The wear volume under different hardness matches is shown in Figure 17. As the surface hardness of 42CrMo4 increases, the wear volume of the friction pair significantly decreases. It can be observed that improving the hardness match can greatly enhance the wear resistance of the specimens. The influence of hardness matching on wear volume decreases monotonically as the hardness increases. Two working conditions, namely 44–60 HRC and 60–60 HRC hardness matches, were selected for analysis, and the experimental data for the two sets showed significant differences, making them representative. The surface topography under different hardness matches is depicted in Figure 18.

Based on the three-dimensional wear topography under the condition of 44 HRC hardness, it can be observed that the maximum depth of the wear track reaches 20 μm. The wear is more severe in the middle region, while the depths of the wear tracks on the sides are less than 10 μm. Combined with Figure 18a, under lower hardness conditions, the primary wear mechanism is plowing accompanied by adhesive wear. Under the condition of 60 HRC hardness, the maximum depth of the wear track is only 4 μm. Under higher hardness matching, the friction pair of 42CrMo4/17NiCrMo6-4 exhibits significantly lower $S_{q}$ and wear volume compared with the specimens with lower hardness matching, indicating that higher hardness is advantageous in reducing wear. This is because as hardness increases, the material’s ability to resist external indentation and scratching is enhanced, leading to improved wear resistance and a reduction in wear volume.

3. The influence of hardness matching on the surface microstructure

**Figure 19:**
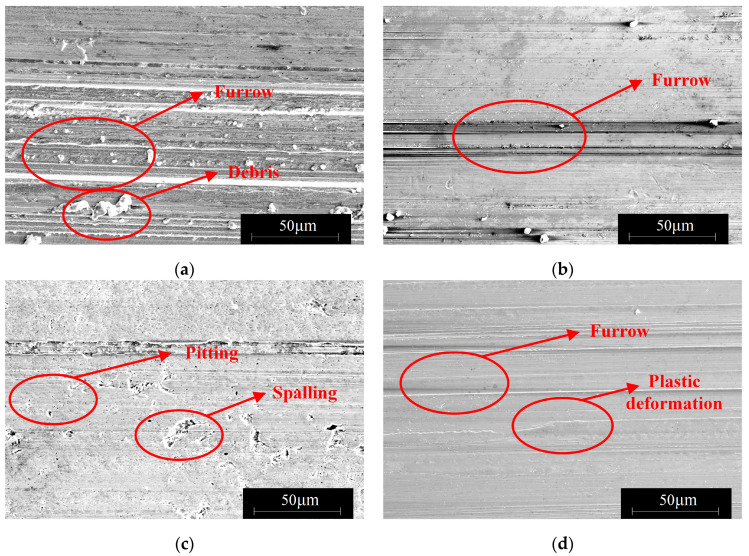
The surface microstructure under different hardness matching. (**a**) 17NiCrMo6-4 with 44–60 HRC; (**b**) 42CrMo4 with 44–60 HRC; (**c**) 17NiCrMo6-4 with 60–60 HRC; (**d**) 42CrMo4 with 60–60 HRC.

Surface microstructures under different hardness matches, as captured by scanning electron microscopy (SEM), are shown in Figure 19. It can be observed that when 42CrMo4 has a hardness of 44 HRC, the wear tracks are deeper, and the presence of wear debris can be seen on both 42CrMo4/17NiCrMo6-4. This type of wear debris is caused by micro-cutting action. When the attack angle of the third body between the friction pair exceeds a certain value, it transitions from plowing to cutting action. As the attack angle decreases, the cutting effect disappears [33,34]. Therefore, wear debris can be observed on the worn surface, and it cannot be easily removed by ultrasonic cleaning. Moreover, there is also significant abrasive wear on 17NiCrMo6-4. The results indicate that under conditions where there is a significant difference in hardness between the friction pair, micro-plowing, and micro-cutting are more likely to occur, leading to an increased friction coefficient and wear volume. 

**Figure 20:**
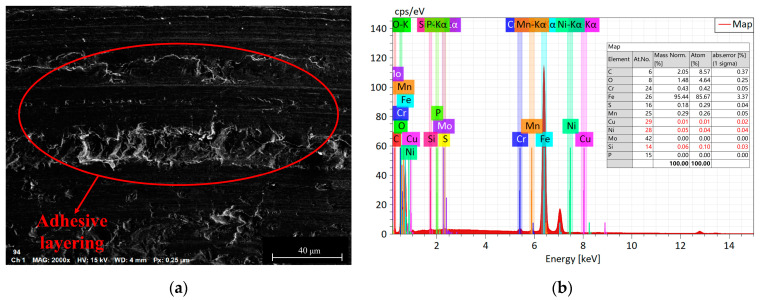
Wear morphologies and EDS diagrams of 42CrMo4 with 44–60 HRC. (**a**) the wear morphologies of 42CrMo4; (**b**) EDS diagrams of 42CrMo4.

When hardness is increased, Figure 19d shows that there is a small number of plowing grooves and adhesive wear on 42CrMo4, as shown in Figure 20. Combined with Figure 18a, this is because lower-hardness materials exhibit higher plasticity, making them more prone to adhesive wear. It becomes more difficult for the friction pair to penetrate the surface of the test specimen, resulting in reduced abrasive wear. However, because of the difficulty of removing material from the surface, 17NiCrMo6-4 is continuously subjected to cyclic loading, which can lead to fatigue wear after multiple stress cycles. Based on the micrographs of 17NiCrMo6-4 under high hardness matching conditions, fatigue spalling and fatigue pitting can be observed. In summary, under the experimental conditions, the higher hardness specimen exhibits better friction reduction and wear resistance. As the surface hardness of 42CrMo4 increases, not only does the wear resistance of the material itself improve, but it also enhances the wear resistance of the mating material. This aligns the wear resistance of the two gear materials, preventing one gear from wearing significantly and deteriorating the meshing performance, thus improving the service life of the gear pair.

## 4. Conclusions

This study primarily analyzes the extent to which different factors affect wear performance. It also examines the main factors that influence the friction and wear performance of gear materials, namely hardness matching, and load, and analyzes the types of wear and wear mechanisms under different operating conditions. The main conclusions are as follows:

(1) Hardness matching has the most significant impact on the wear resistance and friction reduction of the friction pair, followed by loads. Increasing the hardness of 42CrMo4 can reduce the friction coefficient and wear volume of the friction pair. The highest surface hardness, compared with the lowest surface hardness of the 42CrMo4 material, resulted in reductions of 21.5% and 87.2% in the friction coefficient and wear volume, respectively. At low hardness, the main failure mode is abrasive wear. At high hardness, the predominant wear type is still abrasive wear, but 17NiCrMo6-4 experiences more severe fatigue wear.
(2) The friction coefficient initially decreases and then stabilizes with increasing load, while the wear volume initially increases and then decreases with increasing load. The wear type transitions from abrasive wear at low loads to a synergistic effect of abrasive and adhesive wear at high loads. With an increase in contact stress, the adhesive layer formation effectively prevents further wear, resulting in a decrease in wear volume.
(3) Under all lubrication conditions, lubricating grease provides sufficient lubrication for the friction pair. The specific changes in friction coefficient and wear volume may be influenced by the interaction of other factors. The criterion for selecting lubricant quantity is to ensure the presence of uniform lubricating grease in the friction system. Excessive lubricant does not improve the wear performance of the friction pair.

## Figures and Tables

**Table 1:** Chemical composition of the upper specimen of 17NiCrMo6-4.

Element	Fe	Ni	Cr	Mo	C	Si	Mn
content%	rest	1.6	1.5	0.30	0.18	<0.40	0.50

**Table 2:** Chemical composition of the lower specimen of 42CrMo4.

Element	Fe	Cr	Mo	C	Mn	Si	Cu
content%	rest	1.2	0.25	0.40	0.80	0.30	0.35

**Table 3:** Grease characteristics of AG14-61.

Production Data	AG14-61
Chemical composition, thickener	aluminum complex soap
Chemical composition, type of oil	synthetic hydrocarbon oil
Solid lubricants, percentage	approx. 20% by weight
Lower service temperature	−50 °C/−58 ℉
Upper service temperature	120 °C/248 ℉
Color space	white
Texture	homogeneous
Worked penetration, DIN ISO 2137, 25 °C, lower limit value	360 × 0.1 mm
Worked penetration, DIN ISO 2137, 25 °C, upper limit value	360 × 0.1 mm
Kinematic viscosity of the base oil, DIN 51,562 pt. 01/ASTM D-445/ASTM D 7042, 40 °C	approx. 65 ${\mathrm{mm}}^{2}/s$
FZG scuffing test, based on DIN ISO 14,635, A/2,76/room temperature, scuffing load stage	≥12
Water resistance, DIN 51,807 pt. 01, 3 h/90 °C, rating	0–90
Drop point, DIN ISO 2176, IP 396	≥180 °C
Flow pressure of lubricating greases, DIN 51,805, test temperature: −50 °C	≤1400 mbar

**Table 4:** Significance analysis design parameters.

Factor	Level				
	1	2	3	4	5
Load/N	400	600	800	1000	1200
Speed/r/min	50	80	110	140	170
Hardness matching/HRC	60–44	60–48	60–52	60–56	60–60
Lubrication/mm	0.5	1	1.5	2	2.5

**Table 5:** Experiment 1: Single-factor design parameters.

Factor	Level				
	1	2	3	4	5
Load/N	400	600	800	1000	1200
Speed/r/min	90				
Hardness matching/HRC	60–60				
Lubrication/mm	1.5				

**Table 6:** Experiment 2: Single-factor design parameters.

Factor	Level				
	1	2	3	4	5
Load/N	800				
Speed/r/min	90				
Hardness matching/HRC	60–44	60–48	60–52	60–56	60–60
Lubrication/mm	1.5				

## Data Availability

The data presented in this study are available on request from the corresponding author. The data are not publicly available due to the data confidentiality requirements of the company where the testing equipment is located.
